# Revealing the secrets of Blue Zones

**DOI:** 10.3389/fphar.2024.1428111

**Published:** 2024-12-12

**Authors:** N. Nuray Ulusu

**Affiliations:** ^1^ Department of Medical Biochemistry, School of Medicine, Koc University, Istanbul, Türkiye; ^2^ Koç University Research Center for Translational Medicine (KUTTAM), Istanbul, Türkiye

**Keywords:** Blue Zones, glucose-6-phosphate dehydrogenase, senescence, glutathione, oxidative stress

## Abstract

Aging is influenced by cellular senescence mechanisms that are associated with oxidative stress. Oxidative stress is the imbalance between antioxidants and free radicals. This imbalance affects enzyme activities and causes mitochondrial dysfunction. It also slows down cellular energy production and disrupts cellular homeostasis. Additionally, oxidative stress stimulates inflammation, increases the number of point mutations, and alters intercellular communication. It can lead to epigenetic alterations, genomic instability, telomere attrition, and loss of proteostasis. Ultimately, these factors contribute to aging and the development of chronic diseases. Glucose-6-phosphate dehydrogenase (G6PD) is an antioxidant enzyme that protects cells from oxidative and nitrosative damage. It helps restore redox balance, preserve macromolecule function, and rescue cells from cellular senescence, autophagy, and stress-induced apoptosis. G6PD is considered an anti-senescence enzyme. The World Health Organization classifies G6PD variants into five groups based on the enzyme’s residual activity. The first four classes are categorized according to the degree of G6PD deficiency, while the fifth class includes variants with enzyme activities greater than normal. Increased G6PD activity does not exhibit clinical manifestations. Consequently, the full spectrum of mutations and the prevalence of increased G6PD activity in the population remain unknown. The world’s oldest and healthiest people live in Blue Zones. These comprise isolated populations, and there may be a geographic prevalence of high-activity G6PD variants that protect against oxidative stress-induced senescence. To uncover the secret of centenarians’ longevity, additional research is needed to determine whether the hidden factor is the increased activity of the G6PD enzyme.

## Introduction

Senescence is the process that causes aging and results from a lifetime of exposure to various stresses, including reactive oxygen and nitrogen species (RONS) (Calcinotto et al., 2019). Oxidative stress is the imbalance between the production and elimination of RONS that can induce senescence and age-related pathologies (Nousis et al., 2023). Aging and senescence are associated with multiple biological changes. These include loss of protein homeostasis, DNA damage, lysosomal dysfunction, and epigenetic alterations (Wyss-Coray, 2016). Chronic inflammation, increased genomic instability, altered metabolism, and cellular waste accumulation also play crucial roles (Daniele et al., 2018). Additional factors include changes in microbiota composition, increased gut permeability (Ferrucci and Fabbri, 2018), and dysfunctional mitochondria, leading to decreased energy production (Guo et al., 2023). Other key aspects are the deregulation of nutrient-sensing and damaged telomeres. Moreover, decreased NAD^+^ concentration, increased NAD^+^ consumption, and prolonged PARP activation contribute to aging (Akbari et al., 2019). Decreased enzyme activities and alterations in enzyme kinetic mechanisms have also been observed in aging (Ulusu et al., 2005; Ulusu and Tandogan, 2006; García-Domínguez et al., 2022). Furthermore, many of these molecular mechanisms are triggered by increased concentrations of RONS and decreased levels of radical scavenging molecules, such as glutathione (GSH), or by a decreased GSH/GSSG ratio (López-Navarro et al., 2020). NADP-reducing enzymes, particularly glucose-6-phosphate dehydrogenase (G6PD), and enzymes that depend on GSH metabolism play fundamental roles in preventing oxidative stress. They help maintain the redox status of cells, which is essential for cell survival and influences aging (Akbay et al., 2004; Stanton, 2012; Gök et al., 2016; Ulusu et al., 2017; Dore et al., 2021; Chen et al., 2022). G6PD-deficient cells exhibit growth retardation (Cheng et al., 2004) and increased accumulation of oxidative DNA damage. Additionally, these cells show increased sensitivity to oxidant-induced senescence (Wu et al., 2009), metabolic alterations, and cell death (Ho et al., 2013). Deficiencies in glutathione synthesis are associated with oxidative stress, as well as with normal aging and senescence (Sekhar et al., 2011; Aoyama and Nakaki, 2013). Antioxidants and radical scavenging enzymes can prevent RONS-induced cellular damage and are needed for a good healthy life, including G6PD, which protects from radicals and is one of the key factors in increasing lifespan (Nóbrega-Pereira et al., 2016; Warraich et al., 2020).

A healthy life is the most valuable asset in the aging population (Nivestam et al., 2020). Okinawa in Japan, the island of Ikaria in Greece, the island of Sardinia in Italy, the Nicoya Peninsula in Costa Rica, and finally, Loma Linda, California, in the United States, are the geographic regions where people live much longer than average in good health. These geographic areas are named “Blue Zones” and are accepted as the world’s healthiest places, with the highest percentage of centenarians living in these areas (Heath et al., 2022; Pes et al., 2022). The geographically defined areas are Loma Linda, CA, United States; Nicoya, Costa Rica; Sardinia, Italy; Ikaria, Greece; and Okinawa, Japan (Buettner and Skemp, 2016). This review discusses whether the increased activity of the glucose-6-phosphate dehydrogenase enzyme is the key to a long and healthy life in Blue Zones.

## Glucose-6-phosphate dehydrogenase

The G6PD (EC 1.1.1.49) enzyme is the first and regulatory enzyme of the pentose phosphate pathway (PPP) (Tiwari M, 2017). The G6PD enzyme is present in the cytosol and mitochondria of all forms of life except Archaea (Campbell and Bernofsky, 1979; NOTARO et al., 2000; Aydemir and Ulusu, 2020b). Glucose-6-phosphate (G6P) is the substrate of the G6PD enzyme (Pelley, 2012; Aydemir and Ulusu, 2023). G6P is the critical substrate in carbohydrate metabolism and connects glycolysis, the PPP, glycogen metabolism, lipogenesis, and the hexosamine pathway (Rajas et al., 2019). G6P can enter glycolysis to supply cellular energy via the phosphohexose isomerase enzyme. Alternatively, it can contribute to the synthesis of glucuronic acid via phosphoglucomutase enzymes, which is important for the detoxification of metabolites and xenobiotics. G6P can also be stored as glycogen, serving as a primary energy source. Additionally, it can be used in the synthesis of amino sugars or the PPP for the production of sugar phosphates and ribose-5-phosphate. Ribose-5-phosphate is essential for RNA and DNA synthesis and also reduces NADP^+^ to NADPH^+^ in humans (Stincone et al., 2015; Chen et al., 2019; Ho et al., 2019; Karlstaedt et al., 2020; Milanesi et al., 2020; Chandel, 2021). NADPH is involved in anabolic reactions, including fatty acid, cholesterol, and amino acid synthesis. It also plays a crucial role in reducing GSH and thioredoxin, which is important for detoxifying RONS. Additionally, NADPH helps protect against metabolic stress, oxidative damage, and the generation of RONS (Chandel, 2021; García-Domínguez et al., 2022). NADPH is also involved in the biosynthesis of deoxyribonucleotides and coenzymes, such as cytochrome P450, which plays a role in the oxidation and reduction reactions of xenobiotics (Guengerich, 2007; Luzzatto et al., 2020).

The substrates and products of the G6PD enzyme can be used in many enzymatic reactions and play fundamental roles in cellular metabolism (Stanton, 2012; Ahamed et al., 2023). G6PD is under complex regulatory control, with translational and posttranslational modifications, including phosphorylation, acetylation, glycosylation, ubiquitination, and glutarylation, playing a critical role in regulating the enzyme’s activity (Meng et al., 2022).

In the majority of cancer types, G6PD is overexpressed to support cancer cell survival, growth, proliferation, metastasis, and invasion (Stanton, 2012; Song et al., 2022). G6PD is considered a potential diagnostic marker and has been found to be increased in certain cancers (Zheng et al., 2023). G6PD can reprogram cellular metabolism, cell growth, angiogenesis, proliferative signaling, and resistance to cell death (Zhang et al., 2014; Yang et al., 2019; Ahamed et al., 2023). Inhibition of G6PD induces autophagy through endoplasmic reticulum stress, and it has been reported that cancer therapy may be more effective when G6PD inhibitors, such as polydatin, are used in conjunction with the treatment (Mele et al., 2019). Inhibiting the G6PD enzyme is crucial as it can trigger extrinsic apoptosis, one of the most common mechanisms of cell death (Yang et al., 2019). Inhibition of autophagy and high expression of the G6PD enzyme are observed in cancer patients (Yang et al., 2024). The GLUT1/aldolase B/G6PD axis induces chemotherapy resistance and is considered one of the therapeutic targets in cancer (Li et al., 2023).

### Glucose-6-phosphate dehydrogenase deficiency

G6PD enzyme deficiency is the most common X-linked recessive enzyme deficiency affecting erythrocytes (Luzzatto et al., 2016). G6PD deficiency is more common in males. It is estimated that over 400–500 million people are affected by this condition (Tripathi et al., 2019; Manco et al., 2022; Kasturiarachi et al., 2024). G6PD-deficient individuals are mostly asymptomatic; however, this enzyme deficiency can lead to acute or chronic hemolysis, hemolytic jaundice, bilirubin-induced neurological dysfunction, kernicterus, cerebral palsy, and even death in both newborns and adults (Lee et al., 2022; Al Blewi et al., 2023). G6PD deficiency causes the oxidative stress-induced denaturation of hemoglobin molecules, forming Heinz bodies. These Heinz bodies damage the erythrocyte membrane and result in hemolytic anemia (Herman, 2024). Factors that induce oxidative stress can cause hemolytic anemia, and the extent of hemolysis is directly related to the level of residual G6PD enzyme activity (Li et al., 2021). The other common symptoms of G6PD enzyme deficiency are fatigue, back and abdominal pain, hemoglobinuria (Belfield and Tichy, 2018), and dark urine (Li et al., 2024).

Erythrocyte hemolysis and thrombosis due to G6PD deficiency are risk factors for deep vein thrombosis (Thompson et al., 2013), viral infections (Aydemir and Ulusu, 2020a; 2021; Aydemir et al., 2021), neurological diseases (Ho et al., 2007; Ulusu, 2015; Aydemir and Ulusu, 2020b), and cardiovascular diseases (CVDs) (Dore et al., 2021). G6PD deficiency has negative effects on cellular growth, embryonic development, and cellular signaling (Ho et al., 2007).

G6PD deficiency overlaps with malaria endemicity (Mbanefo et al., 2017) and, the severe Mediterranean variant of G6PD deficiency may have protective effects against *Plasmodium vivax* malaria (Uyoga et al., 2015; Awab et al., 2021).

The G6PD enzyme plays a key role in nucleotide and DNA synthesis, DNA repair, and the cell cycle. The G6PD enzyme is upregulated in various cancers. Additionally, G6PD deficiency can have a beneficial effect on cancer treatment and help reverse chemotherapeutic resistance (Song et al., 2022). In cancer cells, the increased activity of G6PD is driven by the reprogramming of the pentose phosphate pathway. This reprogramming is regulated by G6PD activators, oncogenic signaling pathways, and metabolic regulators such as the hypoxia-inducible factor (HIF), phosphoinositide 3-kinases (PI3Ks), mTORC1, KRAS, and NRF2. Additionally, G6PD inhibitors like PTEN, p53, and AMPK are often mutated in cancer. The regulation of metabolic pathways and enzymes in cancer cells differs fundamentally from that in normal cells (Yang et al., 2021; Chu et al., 2023). On the other hand, G6PD-deficient individuals can also have malignancies, and some anticancer drugs can trigger hemolysis. In a case report, a G6PD-deficient patient with breast cancer, despite being at risk for hemolytic anemia and undergoing chemotherapy, endocrine treatment, and radiotherapy, showed no variations in hemoglobin levels or evidence of hemolysis in laboratory results (Chung et al., 2019). G6PD deficiency leads to impaired cell growth and cellular senescence associated with oxidative DNA damage; however, no telomere shortening has been observed (Wu et al., 2009). G6PD deficiency leads to the activation of reactive oxygen species (ROS), TGF-β, and the NADPH oxidase (NOX) system, which regulate immune function, proliferation, cancer, and vascular dysfunction and contribute to cardiovascular disease (CVD) (Parsanathan and Jain, 2020). G6PD deficiency leads to the increased production of ROS, which can have adverse effects and weaken the immune system. It is also a risk factor for autoimmune thyroid disease (Dore et al., 2023).

### Acquired G6PD deficiency

It is well known that G6PD deficiency is a genetic disorder, with the G6PD gene located on the Xq28 band. However, certain endocrine and metabolic conditions, such as hyperaldosteronism and diabetes, can inhibit the G6PD enzyme without any genetic defect. This is referred to as acquired G6PD deficiency (Pes and Dore, 2022). Studies in experimental type-2 diabetes models have shown that diabetes decreases G6PD enzyme activity. In a type-2 diabetes model, increased levels of cytokines TNF-α, IL-6, and IL-1β were linked to decreased G6PD activity due to NF-κB activation and endoplasmic reticulum stress. Additionally, decreased G6PD enzyme activity was restored using a combination of resveratrol and vitamin D in an experimental diabetic rat model (Anapali et al., 2022). The decrease in G6PD activity caused by diabetes-induced oxidative stress was investigated, and G6PD enzyme activity was restored using the powerful antioxidants SMe1EC2 (Ulusu et al., 2017), timolol (Ulusu et al., 2019), and selenium (Can et al., 2005).

### Favism

Favism is a common form of hemolytic anemia associated with 14 different G6PD mutations and has been studied across various geographic regions (Reading et al., 2016; Luzzatto and Arese, 2018). Favism is a condition associated with G6PD deficiency, but not all individuals with G6PD deficiency experience favism (Chandel, 2021). Fava beans contain divicine and isouramil, which can deplete the glutathione content of erythrocytes, leading to their destruction (Chevion et al., 1982). The clinical features of favism are similar to those of other G6PD enzyme deficiencies, with severity varying based on the type of mutation and age (Beretta et al., 2023). Favism can lead to the formation of Heinz bodies (Lee et al., 2022), abdominal pain (Diegues et al., 2022), malaise, splenomegaly, systolic murmur (Cahill and Ley, 1962), hepatomegaly (Emanuel and Schoenfeld, 1961), and even death (Mentzer and Collier, 1975). In a study of cell aging, the erythrocyte aging pathways were examined in both normal individuals and those with favism. It was found that G6PD-deficient erythrocytes exhibit a unique metabolic regulation and energy consumption mechanism specific to favism pathology. Surprisingly, erythrocytes from patients with favism were more resistant to extreme stress conditions than those from healthy individuals (Dinarelli et al., 2022).

### Glucose-6-phosphate dehydrogenase variants

A total of 230 G6PD genetic variants (also called mutations) have been described clinically (Pfeffer et al., 2022), and some of these variants cause significant phenotypic severity depending on the mutation location (Lee et al., 2022). The World Health Organization (WHO) has classified G6PD variants into five groups based on the severity of the deficiency and the enzyme’s biochemical properties. The first group is associated with chronic non-spherocytic anemia (CNSHA), which is considered the most severe form. The second group includes individuals with severe deficiency, with less than 10% of normal G6PD activity. The third group comprises those with moderate deficiency, with 10%–60% of normal activity, while individuals with normal G6PD activity are placed in the fourth group (Glucose-6-phosphate dehydrogenase deficiency. WHO Working Group, 1989; Ezz El-Deen et al., 2013). Most G6PD-deficient individuals are classified under the second and third classes, with the prevalence of different mutations varying from 1% to 70% across different geographic areas (Mason et al., 2007). In a virtual meeting in 2022, the WHO updated the G6PD classification, introducing new categories: classes A, B, C, and U. Class A, a rare chronic condition, corresponds to the previous class I, where the median G6PD activity is less than 20%, associated with CNSHA. Class B includes variants that trigger acute episodes, with a median G6PD activity of less than 45%. Class C refers to the group without hemolysis, where G6PD activity ranges from 60% to 150%. Class U encompasses novel variants with uncertain clinical significance (World Health Organization, 2022).

The prevalence of G6PD deficiency varies among different populations (DePina et al., 2020). G6PD deficiency exhibits a wide geographic distribution (Lee et al., 2022). G6PD deficiency is rare in Japan, with approximately 21 G6PD variants being characterized. Some Japanese variants are classified as class 1, including Tokyo, Yokohama, Yamaguchi, Wakayama, Akita, Heian, Kyoto, Tokushima, Ogikubo, Kurume, and Fukushima. Variants classified as class 2 include Hofu, B(−) Chinese, Ube, Konan, and Kamiube. The Kiwa variant belongs to class 3 (Miwa, 1980). Nakashima et al. demonstrated that G6PD deficiency is very rare, identifying it in only 5 out of 6,120 Japanese men (Nakashima K, 1977). G6PD deficiency is exceedingly rare in Latin America. In Costa Rica, the prevalence of G6PD deficiency, particularly for classes 1 and 2, is notably low. Puerto Limón has a rare incidence of class 1 deficiency, while class 2 variants are observed in Santamaría. Overall, G6PD deficiency is very uncommon in these regions, suggesting that the prevalence in Nicoya might be close to zero (Monteiro et al., 2014). G6PD deficiency is more common in continental Greece, but it is rare in the Greek islands. The lowest frequency is recorded in the Aegean and Ionian islands, including Ikaria, with a prevalence of 2.9% (Stamatoyannopoulos et al., 1966). The Blue Zone pertains specifically to the central region of Sardinia, not the entire island. Among the limited studies of G6PD deficiency across Sardinia’s municipalities, one study found that G6PD deficiency is less prevalent in the central area, which corresponds to the Blue Zone than in the peripheral areas (Pes GM et al., 2017). This suggests that the observed longevity in the Blue Zone may be associated with a lower prevalence of G6PD deficiency. Some studies from Sardinia do not support the idea that high G6PD activity extends the human lifespan. For instance, it has been suggested that G6PD inhibition may slow the development of age-related diseases. Schwartz and Pashko proposed that dehydroepiandrosterone analogs could be valuable in slowing the progression of various age-related conditions (Schwartz and Pashko, 2004). A review from Italy also discussed the advantages and disadvantages of G6PD, suggesting that G6PD deficiency may provide benefits with respect to malaria, cancer development, and coronary disease and may also have positive effects on longevity (Manganelli et al., 2013). Another study suggests that G6PD deficiency is a factor influencing longevity in men in Sardinia (Poulain et al., 2011).

The highest prevalence of G6PD variants is found in sub-Saharan Africa (Nkhoma et al., 2009), followed by the Arabian Peninsula, Central and South Asia (Howes et al., 2012), and Mediterranean Europe (Lee et al., 2022). On the other hand, the highest incidence of G6PD deficiency in the world is observed in the Kurdish Jewish population, where 70% of male subjects are affected. The most common variant in this group is G6PD Mediterranean (Oppenheim et al., 1993). G6PD deficiency is most commonly found in Africa, followed by Asia and the Mediterranean region (Frank JE, 2005). The most common G6PD variant is A-, which is prevalent among Ethiopians. Three SNPs have been detected in this variant: the most common mutation is A376G, with the other mutations being G267 + 119/T and G116A (Lo et al., 2019).

Class 5 mutations result in increased G6PD enzyme activity. The clinical features of these variants are distinctly different from those of other classes. Increased enzyme activity generally presents no clinical symptoms or signs and is typically identified through biochemical changes (Glucose-6-phosphate dehydrogenase deficiency. WHO Working Group, 1989). For instance, a reduction in GSH, a crucial antioxidant, can occur. An increased concentration of GSH may thus play a role in various detoxification reactions and antioxidant defense systems. Therefore, an increased concentration of GSH would contribute to various detoxification reactions and antioxidant defense systems (Ulusu et al., 2003; Saitoh et al., 2011; Aydemir et al., 2020). GSH is correlated with longevity; increased concentrations of glutathione help reduce oxidative stress and its damaging effects (Detcheverry et al., 2023).

### Glucose-6-phosphate dehydrogenase and NADP^+^

NAD^+^ and NADP^+^ are different coenzymes with distinct roles in metabolism. NAD^+^ can be converted into NADP^+^ through a reaction catalyzed by the enzyme NAD^+^ kinase. Conversely, the enzyme NADP^+^ phosphatase can convert NADP^+^ back into NAD^+^. The activities of NAD^+^ kinase and NADP^+^ phosphatase regulate the intracellular balance between these two molecules according to the cell’s metabolic needs (Kawai and Murata, 2008). It has been reported that NAD^+^ concentration decreases with aging, and maintaining higher levels of NAD^+^ is associated with a longer lifespan (Schultz and Sinclair, 2016). Since almost all NADP^+^ is synthesized from NAD^+^ through cytosolic and mitochondrial kinases, a decrease in NAD^+^ levels with aging would also lead to a reduction in NADP^+^ concentration (Bradshaw, 2019). NADPH plays two crucial roles in aging. First, it helps maintain redox homeostasis by protecting against oxidative stress. Second, NADPH is critical for various cellular functions, including the cell cycle and anabolic reactions. Consequently, NADPH homeostasis is tightly regulated by diverse signaling pathways and specific metabolic enzymes (Ju et al., 2020). An experimental *Drosophila* model demonstrated that the overexpression of the G6PD enzyme increased the levels of NADPH, NADH, and the GSH/GSSG ratio. These results are associated with enhanced biosynthetic and antioxidant capabilities in the organism and correlate with lifespan extension (Legan et al., 2008). A similar study was conducted on transgenic mice with moderate overexpression of the G6PD enzyme. The study found that higher levels of NADPH and reduced ROS-related damage were associated with an increased lifespan (Nóbrega-Pereira S, 2016). Therefore, the G6PD enzyme can be considered an anti-senescence enzyme due to its direct and indirect effects on cellular functions (Stanton, 2012).

### Glucose-6-phosphate dehydrogenase and glutathione

GSH is a key antioxidant molecule that plays a crucial role in the cytosol, nucleus, endoplasmic reticulum, and mitochondria and is involved in many antioxidant pathways. This tripeptide is primarily synthesized in the cytosol through enzymatic reactions (Marí et al., 2009). It is then distributed to various organelles via Na^+^ -dependent and Na^+^ -independent pathways. The liver plays a pivotal role in the inter-organ transport of GSH (Vašková et al., 2023). Glutathione serves as a marker of the redox state in aging processes, disease, and cell death (Vašková et al., 2023; Tandogan and Ulusu, 2011). It also plays a central role in the detoxification of xenobiotics and drugs. Glutathione-S-conjugates are water-soluble and readily excreted from cells and the body. However, some glutathione conjugates of drugs or chemicals can transform into thioethers, which can become more active and toxic than the parent compound (Potęga, 2022). During the detoxification process, either through non-enzymatic conjugation or reactions catalyzed by glutathione-S-transferase (GST) enzymes, GSH can contribute to drug resistance (Mazari et al., 2023). Under oxidative stress conditions, GSH is oxidized to form glutathione disulfide (GSSG) from two GSH molecules. Subsequently, the enzyme glutathione reductase (GR) uses NADPH to convert GSSG back to its reduced form (Aydemir et al., 2019; Chen et al., 2024). Furthermore, when oxidative stress is excessively high, the concentration of GSSG increases while GSH levels decrease, leading to a reduced GSH-to-GSSG ratio. This GSH/GSSG ratio reflects the redox capacity of the cell (Tandogan and Ulusu, 2010; Chen et al., 2024). Mitochondria are the major source of ROS, and an imbalance between ROS and mitochondrial GSH (mGSH) can lead to cellular dysfunction, lipid peroxidation, mitochondrial DNA mutations or deletions, and various forms of cell death, such as apoptosis, necroptosis, and ferroptosis. This imbalance may contribute to the development of disease (Chen et al., 2024). Specific increases in RONS levels reduce cell resistance to stress, leading to macromolecular damage, altered gene expression, and the development of aging and age-related diseases and pathologies (Davalli et al., 2016). ROS can activate mitogenic cell signaling pathways such as PI3K/AKT/mTOR and MAPK. This activation contributes to epithelial-to-mesenchymal transition in cancer cells, increases the expression of matrix metalloproteinases, leads to extracellular matrix degradation, induces angiogenesis, and facilitates cancer cell metastasis (Wang et al., 2021).

## The Blue Zones: Where people live healthy and long

The Blue Zones, Loma Linda, CA (United States); Okinawa (Japan); Ikaria (Greece); Sardinia (Italy); and the Nicoya Peninsula (Costa Rica), are recognized as geographic regions where people live beyond 90 years without significant chronic disease (Pes et al., 2022). The lifestyles, health status, and genetic markers of the long-lived populations on Sardinia and Ikaria islands are very similar (Poulain et al., 2021). Notably, there is an unusually high number of elderly people living in the mountainous regions of Sardinia (Poulain et al., 2004). Islands are closed populations, and there may be a high rate of inbreeding, which can increase the risk of disease variants n, for instance, late-onset Alzheimer’s disease (Vardarajan et al., 2015). On the other hand, this can also have the advantage of preserving beneficial inherited mutations, such as the class 5 variant of the G6PD enzyme (Glucose-6-phosphate dehydrogenase deficiency. WHO Working Group, 1989).

The common features of Blue Zone populations, such as not smoking, are directly correlated with reduced radical formation and decreased cardiac risk factors (Lakshmanan et al., 2020). However, it is notable that some smokers of both genders are found in Blue Zone populations, particularly in Sardinia and Ikaria (Poulain et al., 2021). Dan Buettner and Sam Skemp studied individuals who have lived over 90 years without chronic disease. These individuals typically do not engage in strength training but maintain a strong sense of purpose and prefer to live with their loved ones, family, and friends. They often reside in faith-based communities. Centenarians are moderate drinkers who enjoy socializing with family and friends. Their diet primarily includes beans, fava beans, black soybeans, and lentils, and they consume meat only about five times a month. Additionally, they practice mindful eating, stopping when they feel 80% full (Buettner and Skemp, 2016). Lifestyle and eating habits are crucial for healthy aging and longevity. The Mediterranean diet and lifestyle are widely considered optimal for providing antioxidants and essential nutrients that support a healthy aging process (Mazza et al., 2021). The Mediterranean diet is among the most extensively studied diets, with well-documented benefits for health. These include reductions in overweight and obesity, cardiovascular disease, cancer, cognitive decline, metabolic syndrome, and diabetes mortality (Guasch-Ferré and Willett, 2021). Sicily is the largest island in the Mediterranean Sea. Although the lifestyle in Sicily is similar to that in Sardinia, the number of centenarians in Sicily is low; therefore, this island is not considered part of the Blue Zones (Spencer Mehalic, 2023). In previous research, researchers selected three villages in the Sicani Mountains of Sicily, a region noted for having the highest number of centenarians in Italy. Out of a population of 10,000, 15 individuals were aged between 100 and 107, which is six times higher than the national average. The longevity of these inhabitants was linked to their adherence to the Mediterranean diet. The number of centenarians was more than 6 times higher than the national population in Sicily. The number of centenarians was 3.56 times higher in Sardinia than in Sicily (Vasto et al., 2012; Angela, 2021). Lifestyles and diets are similar across Italy and its islands, so life expectancy might be expected to be similar. However, it is not; the population of Sardinia has a significantly longer life expectancy (Vasto et al., 2012; Angela, 2021). Italians prefer the Mediterranean diet, which includes fresh fruits and vegetables (Marche et al., 2024). Another notable detail is that centenarians eat fava beans (Buettner and Skemp, 2016). The consumption of fava beans among centenarians in Sardinia suggests that this population does not suffer from favism (Luzzatto et al., 2023).

The class 5 variant of the G6PD enzyme in the human population is more likely to contribute to longevity than the lifestyle of long-living Blue Zone populations. This is due to the increased activity of G6PD, which effectively detoxifies radicals and helps prevent aging. If lifestyle were the sole factor, then populations across the Greek islands, which follow the Mediterranean diet and have similar lifestyles, would show comparable longevity (Kapelios et al., 2017).

Therefore, additional studies are needed to determine whether the high activity of the G6PD enzyme is a key factor in the longevity observed in Blue Zone populations, particularly among those with the class 5 G6PD enzyme variants.

## The possibility of a class 5 G6PD enzyme variant in Blue Zone populations

Genetic and non-genetic factors, such as social and cultural lifestyle factors, unhealthy foods, inflammation, and environmental influences, are key determinants of health and longevity (Castruita et al., 2022). The Mediterranean region includes thousands of islands and extensive coastlines, all of which are characterized by similar Mediterranean diets and lifestyles (Al Wattar et al., 2019). The Mediterranean population shares similar dietary habits, predominantly plant-based, with a focus on meals prepared with extra virgin olive oil (Mattavelli et al., 2022). The Mediterranean lifestyle, including physical activity, sleep quality, and environmental conditions, is remarkably consistent across all Mediterranean islands (Mantzorou et al., 2023). The Mediterranean diet helps prevent chronic degenerative diseases, cancer, diabetes, and cardiovascular disease, and it promotes overall health (Ndlovu et al., 2019). Islands are considered isolated ecosystems and natural laboratories, making them ideal for investigating the relationships between ecology, evolution, aging, and conservation (Matthews and Triantis, 2021). In the Mediterranean region, French wines play a notable role, famously associated with the “French Paradox.” French wines contain strong antioxidants such as resveratrol and various micronutrients that are key to reducing LDL cholesterol in both healthy individuals and those with health conditions, contributing to overall health and longevity. Although the French population follows a Mediterranean diet and lifestyle, French wine consumption offers numerous health benefits. However, no specific region or island in France is included in the Blue Zones (Fragopoulou et al., 2018). Greece and Italy have hundreds of islands. There must be more than two islands that are included in the Blue Zones (Pes et al., 2022). However, there must be specific factors that set these islands apart and contribute to the exceptional longevity and health of their populations in the Mediterranean region (Buettner and Skemp, 2016).

In Loma Linda, CA, the diet differs from the Mediterranean diet and is predominantly vegan or vegetarian, with 27% of the diet consisting of fruits and 33% of vegetables, while meat or fish makes up only 5%. In contrast, the Okinawan diet is rich in sweet potatoes (67%), rice (12%), and other grains (3%). In the Nicoya region, the diet includes 26% whole grains and 24% dairy products (Aliberti et al., 2024). These variations in key nutritional components highlight differences in dietary patterns that influence the lifespan among populations living in the Blue Zones.

Loma Linda, CA, in the United States; Okinawa in Japan; and the Nicoya peninsula in Costa Rica, (Pes et al., 2022) are geographically, nutritionally, and lifestyle-wise distinct from the islands of Ikaria (Greece) and Sardinia (Italy). Therefore, there is no single common environmental factor affecting lifespan and health across all these Blue Zone regions.

One factor that may influence a healthy, long life is genetic variation. The key could be an enhanced system for detoxifying RONS and synthesizing essential macromolecules, as well as regulating the PPP. For example, high activity of the G6PD enzyme might facilitate these processes effectively (Stanton, 2012; Aydemir and Ulusu, 2020b). Increased activity of G6PD enzyme variants can detoxify radicals and xenobiotics, thereby protecting cells from age-related and pro-inflammatory factors (Errigo et al., 2023), such as metabolic stress, age-associated functional decline, and effects on lifespan, and protecting against cellular stress-dependent diseases (García-Domínguez et al., 2022).

All of these data suggest that we need to measure G6PD enzyme activity levels in centenarians to gain deeper insights into their role in longevity. By investigating these levels, we might uncover pivotal factors that not only contribute to their exceptional lifespan but also offer valuable clues for enhancing human health and extending life.

## Conclusion

Every living organism is born, ages, and eventually dies, with the rate of aging being unique to each individual. The variables influencing aging are multifactorial, including environmental factors, individual genetic and epigenetic properties, biochemical and physiological factors, diet, and lifestyle. Oxidative stress is a fundamental factor in age-related changes. There must be a common factor that reduces oxidative stress and promotes health and longevity in the Blue Zone populations. Although individuals in these regions exhibit variations in their diet, lifestyle, and environment, measuring G6PD enzyme activity among them may help uncover the secrets of a healthy and long life. The G6PD enzyme protects cells from oxidative stress by removing free radicals and contributes to the synthesis of biomolecules. Therefore, it can be considered an anti-senescence enzyme. This enzyme helps safeguard cells from oxidative stress-induced damage through its anti-senescence properties. Further research is needed to determine whether the increased activity of G6PD enzyme variants is a key factor in the longevity of Blue Zone populations. Measuring G6PD enzyme activity in centenarians could reveal important insights into the secrets of a long and healthy life.
